# Direct growth of graphene on Ge(100) and Ge(110) via thermal and plasma enhanced CVD

**DOI:** 10.1038/s41598-020-69846-7

**Published:** 2020-07-31

**Authors:** Bilge Bekdüz, Umut Kaya, Moritz Langer, Wolfgang Mertin, Gerd Bacher

**Affiliations:** Werkstoffe der Elektrotechnik and CENIDE, Universität Duisburg-Essen, 47057 Duisburg, Germany

**Keywords:** Materials science, Nanoscience and technology

## Abstract

The integration of graphene into CMOS compatible Ge technology is in particular attractive for optoelectronic devices in the infrared spectral range. Since graphene transfer from metal substrates has detrimental effects on the electrical properties of the graphene film and moreover, leads to severe contamination issues, direct growth of graphene on Ge is highly desirable. In this work, we present recipes for a direct growth of graphene on Ge via thermal chemical vapor deposition (TCVD) and plasma-enhanced chemical vapor deposition (PECVD). We demonstrate that the growth temperature can be reduced by about 200 °C in PECVD with respect to TCVD, where usually growth occurs close to the melting point of Ge. For both, TCVD and PECVD, hexagonal and elongated morphology is observed on Ge(100) and Ge(110), respectively, indicating the dominant role of substrate orientation on the shape of graphene grains. Interestingly, Raman data indicate a compressive strain of ca. − 0.4% of the graphene film fabricated by TCVD, whereas a tensile strain of up to + 1.2% is determined for graphene synthesized via PECVD, regardless the substrate orientation. Supported by Kelvin probe force measurements, we suggest a mechanism that is responsible for graphene formation on Ge and the resulting strain in TCVD and PECVD.

Fabrication of large area graphene is well established on catalytic metal foils, such as copper (Cu)^1−3^, due to the ability to control monolayer formation, easy scalability^4,5^ and high quality of monolayer graphene. Graphene with a charge carrier mobility up to 10,000 cm^2^ V^−1^ s^−1^^6^ and a sheet resistance down to 105 Ω/sq.^7^ has been demonstrated. In order to implement the graphene film into device technology, it must be transferred from the metal foil onto the desired substrate. This transfer process deteriorates the graphene film quality as a result of contamination from metal substrate^8^, etching agent^9^ and supporting polymer film^10,11^ and due to wrinkle formation^12^. This severely limits the use of transferred graphene layers in semiconductor device technology. Hence, direct fabrication of graphene on non-metal substrates like silicon (Si)^13^, germanium (Ge)^14−17^, silicon dioxide (SiO_2_)^18,19^ and sapphire (Al_2_O_3_)^18,19^ has become an alternative route in the last few years.

Similar to Cu, Ge exhibits a self-limiting effect resulting from the low carbon solubility in Ge^20^ and thus, enables monolayer graphene synthesis^21^. The graphene flakes were reported to nucleate via covalent bonds at Ge dimer vacancies on a surface with (100) crystal orientation^22^. Di Gaspare et al.^23^ found a carbon precursor phase, which evolved to graphene domains through a recrystallization process with increasing growth time. By varying the amount of precursor, the morphology of graphene on Ge(100) can be changed from nanoribbons to round shaped flakes^24^. Persichetti et al.^16,25^ suggested that the elevated growth temperature is the critical parameter for reducing the amount of defects in the graphene film, which in turn increases the compressive strain in the film and induces surface faceting of Ge(100). A defect-free monolayer graphene film was demonstrated on a 200 mm Ge/Si wafer with (100) crystal orientation, on which a sheet resistance of 1,500 ± 100 Ω/sq. and a charge carrier mobility of 400 ± 20 cm^2^ V^−1^ s^−1^ were determined^15^. Lee et al.^14^ observed that elongated flakes were formed on Ge(110), which merged into a defect-free (*I*_D_/*I*_G_ < 0.03) graphene film with a charge carrier mobility of 2,560 ± 460 cm^2^ V^−1^ s^−1^ and a sheet resistance between 2 kΩ/sq. and 5 kΩ/sq. The elongated shape of the graphene grains was related to the covalent bonds between edge atoms of Ge(110) and graphene nuclei at the early stage of growth^26^.

The attractiveness of graphene integration into CMOS technology arises from a wide variety of potential applications, including photodetectors and modulators for the NIR regime^27−29^ or passivation layers^17,30^. Hereby, different Ge platforms, like Ge on Si^31,32^ or Ge on insulator^33,34^ are used. It is noteworthy, that until now the synthesis of graphene on Ge has been established close to the melting temperature of Ge (937 °C)^24,35,36^. This leads to fundamental challenges in the process development. At these high temperatures, the doping profile of the functional Ge layer may change and/or Si may diffuse towards the surface in case of Ge on Si, forming SiC during graphene growth^37,38^. While PECVD has the potential to significantly reduce the growth temperature for depositing graphene on Ge, this approach has not yet been shown in literature up to now.

Here, we report on graphene synthesis on Ge at temperatures down to < 760 °C, i.e., almost 200 °C below the melting point of Ge, in a plasma enhanced CVD process. As a reference, TCVD growth is performed at elevated temperatures in the same reactor. By varying growth time and Ge crystal orientation, graphene flake morphology, grain size and strain properties of the films are studied. Based on our data, a model is suggested for graphene formation on Ge by TCVD and PECVD.

## Results and discussion

### Graphene synthesis via TCVD on Ge(100) and Ge(110)

**Figure 1:**
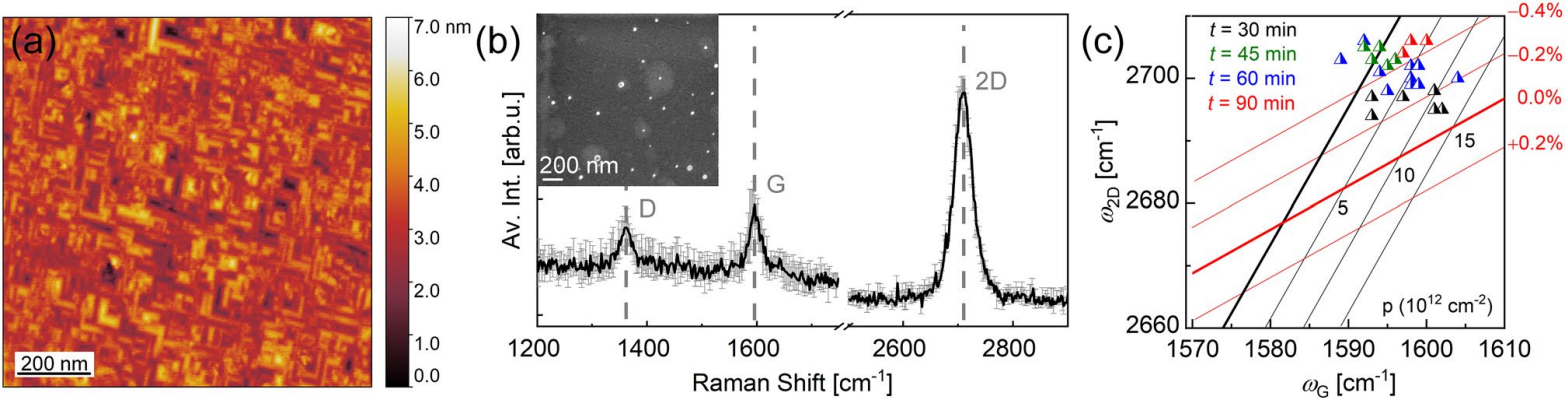
TCVD graphene on Ge(100) grown at 927 °C. (**a**) AFM topography map after graphene synthesis. (**b**) Average Raman spectrum of the sample with a growth time of 90 min, determined from three randomly selected points on the substrate. In the inset, an SEM image of the sample is presented. (**c**) Position of the 2D-peak vs. position of the G-peak for TCVD graphene on Ge(100) fabricated at different growth times (triangles with varied colours). For the Raman spectra of these samples see Figure S2 in SI. The solid lines represent the strain and doping levels according to Ref^44^.

In a first step, graphene was synthesized on Ge(100) with the plasma turned off. The substrate temperature was adjusted close to the melting temperature of Ge. During the growth process 3 sccm of CH_4_, 100 sccm of H_2_ and 450 sccm of Ar were introduced into the chamber at a total pressure of 750 mbar. The topography of the fabricated sample was analyzed by AFM (Fig. 1a). The well-known facet formation of Ge(100) is apparent from the data. Kiraly et al.^39^ found that during the cooling process, graphene drives the Ge(100) surface to reorganize along the (107) plane in order to minimize the surface free energy^40^. By a detailed AFM topography analysis, we extracted an average facet angle of 8.5°, which according to McElhinny et al.^40^ indicates the formation of {107} facets in our samples (see Figure S1 in SI). Furthermore, Grzonka et al.^41^ observed that Ge(100) undergoes faceting exclusively underneath the graphene area. The observed continuous faceting of the sample surface thus indicates the presence of a fully coalesced graphene film.

In Fig. 1b the Raman spectrum of graphene is presented with a scanning electron microscopy (SEM) image in the inset. The Raman spectrum is averaged over Raman spectra from three randomly selected positions on the substrate. The standard deviation is indicated by grey colour. The *I*_2D_/*I*_G_ ratio of 3.9 and the full width half maximum (FWHM) of the 2D-peak of 40 cm^−1^ are ascribed to predominantly monolayer graphene. Thus, we interpret the bright regions on the dark background seen in the SEM image (see inset of Fig. 1b) as multilayer islands on a fully closed monolayer film. Similar to literature^42,43^ some white dots are present in the SEM image, which are most likely related to an oxidation process of Ge as described in a recent study^17^. Obviously, the graphene film is defective with an *I*_D_/*I*_G_ ratio of 0.7. Under similar growth conditions, Scaparro et al.^24^ observed a comparable defect density (*I*_D_/*I*_G_ ratio ~ 0.5 and ~ 1.5 in presence of multilayers), which was reduced by lowering the amount of the precursor.

In order to access strain and doping properties of the fabricated film, the position of the 2D (*ω*_2D_) Raman peak of the graphene is plotted versus the position of the G (*ω*_G_) peak in Fig. 1c for various growth times. SEM images and average Raman spectra of these samples are presented in Figure S2 in SI. After a growth time of 30 min a contrast difference is apparent at roughly 50% of the surface, which is attributed to the surface faceting in presence of graphene. Between 45 min and 60 min of growth time full surface coverage is achieved and multilayers are formed as indicated by a reduction of the 2D to G peak intensity ratio (see Figure S2(c) in SI). At 90 min of growth time, the 2D to G peak intensity ratio increases drastically, which may originate from increased crystallinity of the fabricated film. On the other hand, the D to G peak intensity increases, which is attributed to the increased multilayer formation. The solid lines added to the figure indicate the respective values of strain (red) and doping (black) expected according to Lee et al.^44^. The data for the samples fabricated at growth times of 30 min, 45 min, 60 min and 90 min are presented in black, green, blue and red, respectively. Analyzing the Raman peak positions, a compressive strain between − 0.2% and − 0.4% is determined, slightly rising with increasing growth time. The graphene film shows p-type doping with a doping level up to ~ 10∙10^12^ cm^−2^, not systematically depending on growth time. These findings are in good agreement with literature, where TCVD grown graphene films on Ge show predominantly compressive strain and p-type doping^23,39,45,46^. Pasternak et al.^46^ explained the compressive strain by the different thermal expansion coefficients of graphene (− 6 × 10^−6 ^K^−1^)^47^ and Ge (+ 5.75 × 10^−6 ^K^−1^)^48^. I.e., upon cooling after the TCVD growth the Ge lattice contracts, leading to a compression of the carbon lattice on top due to a (strong) interaction between graphene and Ge. Kiraly et al.^39^ evaluated the strain in graphene fabricated on Ge(100) and Ge(110) and obtained a compressive strain up to − 1.0%. Lukosius et al.^15^ also extracted a compressive strain up to − 0.15% on TCVD graphene fabricated on Ge(100), and estimated a p-type doping level of 2 × 10^13^ cm^−2^, agreeing with our findings.

**Figure 2:**
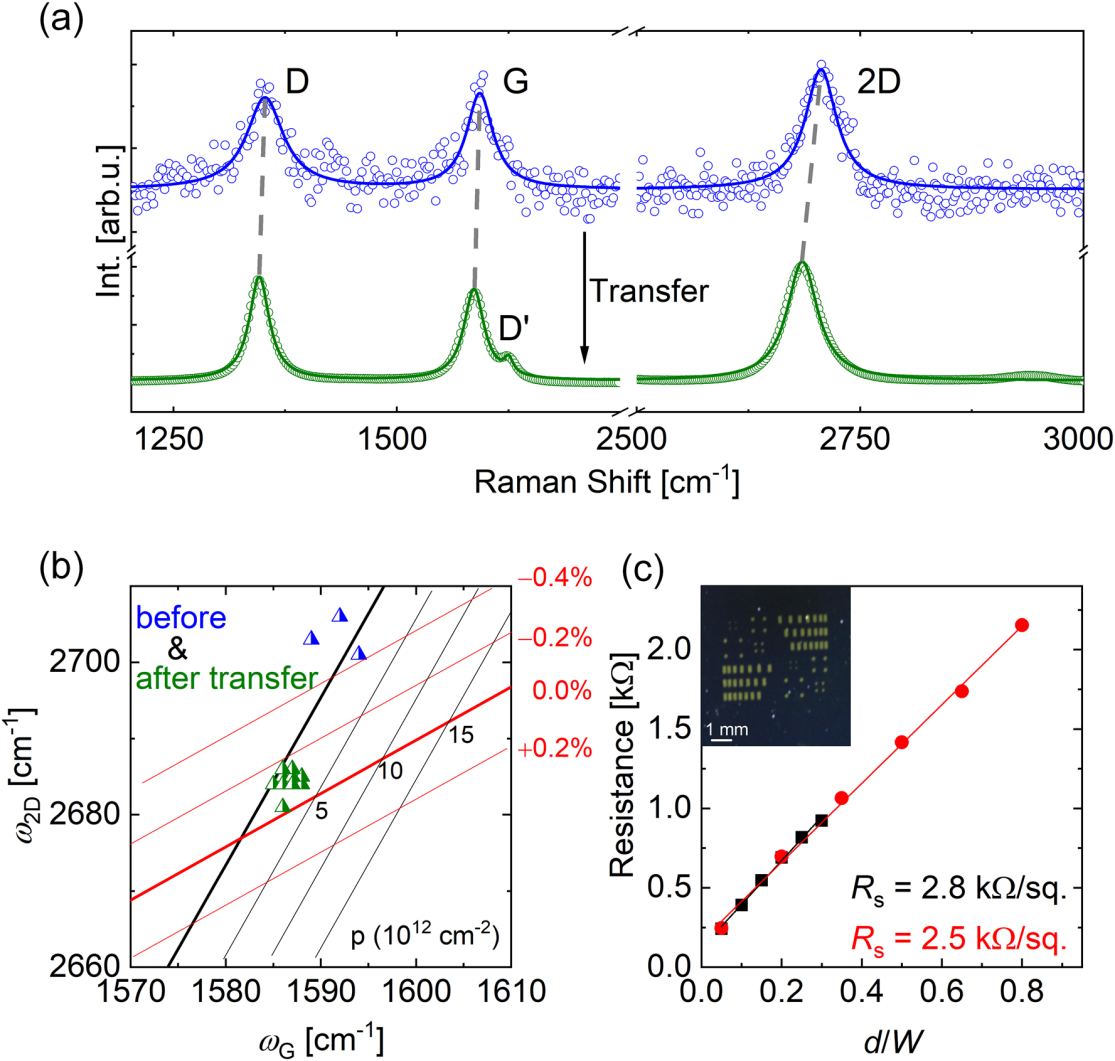
The effect of graphene transfer on the strain properties of the fabricated film. (**a**) Raman spectra of the graphene film on Ge(100) (blue) and after transfer onto Si/SiO_2_ (green). The D, G, D’ and 2D-peaks are indicated in the figure. The solid lines are Lorentzian fit functions. (**b**) Wavenumber of the 2D- vs. wavenumber of the G-peak. Symbols represent the data before (in blue) and after (in green) transfer. Solid lines indicate the expected strain and doping levels according to Ref^44^. (**c**) TLM measurements on a transferred graphene film. The electrical resistance is plotted as a function of the ratio of the distance between two electrodes to the width of the contact pads (*d*/*W*) for two different structures on one sample. An optical image of the measured sample with different TLM structures is depicted in the inset.

In order to investigate the origin of the compressive strain and to assess the electrical properties of the fabricated film, the graphene layer is transferred onto a Si/SiO_2_ substrate by electrochemical delamination (see “Methods” section). Raman spectra of the graphene are shown in Fig. 2a before (blue dots) and after (green dots) transfer. The graphene was fabricated with a growth time of 60 min (see also blue dots in Fig. 1c). The solid lines represent Lorentzian fits of the spectra, and the D-, G- and 2D-peaks are indicated in the figure. Although the Raman spectrum of graphene that is measured on Ge is noisy, the important peaks are evident. After transfer, the D′-peak can be resolved due to the reduced noise. According to literature, the intensity ratio of *I*_D_/*I*_D′_ is related to the type of the defects involved^49^. From our data, a ratio of *I*_D_/*I*_D′_ ≈ 3.8 is extracted, which indicates the dominant role of boundary defects, which may stem from the grain boundaries of the monolayer graphene as well as from the dangling bonds of the multilayer islands. The dashed, grey lines indicate the shift in the peak frequency after transfer. While the positions of the D- and G-peaks vary only slightly, the 2D-peak shifts by 21 cm^−1^ to lower wavenumbers.

Again, the 2D- and G-peak positions are plotted against each other to estimate strain and doping level before (in blue) and after (in green) the electrochemical transfer onto a Si/SiO_2_ substrate (Fig. 2b). Interestingly, the compressive strain of ~  − 0.4% obtained for the graphene film prior to the transfer is reduced to ~  − 0.1% after separation of the film from the Ge wafer and deposition onto the Si/SiO_2_ substrate. This is in line with the explanation given above emphasizing the role of the different thermal expansion coefficients of Ge and graphene and the resulting compressive strain in the graphene layer during the cool-down step. As soon as the interaction between graphene and Ge is lost, graphene relaxes, reducing the compressive strain.

The electrical properties of the graphene film are determined by the transfer-length method (TLM) on a Si/SiO_2_ substrate. The graphene film was fabricated at 927 °C for a growth time of 60 min under CH_4_:H_2_:Ar flows of 5:20:450 sccm at a chamber pressure of 750 mbar. By changing the CH_4_/H_2_ ratio from 5:20 and 5:100 to 3:100, we obtained similar Raman spectra (see SI Figure S3), indicating a comparable film quality within this process window. A TLM mask was evaporated by a shadow-mask technique onto the graphene film (Fig. 2c inset). The resistance is measured between two electrodes of varying distance. Two exemplary measurements are shown in red and black, which are performed on one sample with different TLM scales (see “Methods” section). The solid lines represent linear fits of the measured data. All values are lying perfectly on top of the linear fit, which indicates a good electrical homogeneity of the film, agreeing with the small standard deviation of the average Raman spectrum shown in Fig. 1b. From the slopes of the fit functions, the sheet resistances (*R*_s_) are determined as 2.5 kΩ/sq. and 2.8 kΩ/sq., respectively. In literature the graphene sheet resistance was reported as 1.5 ± 0.1 kΩ/sq. for graphene on Ge(100)^15^ and > 2 kΩ/sq. for graphene on Ge(110)^14^. The marginal deviation in the sheet resistance with respect to literature is attributed to a larger defect density in the fabricated film, as indicated by the D-peak in the Raman spectrum.

**Figure 3:**
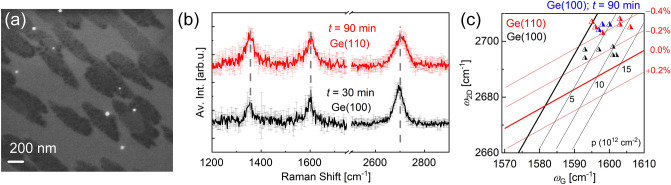
TCVD grown graphene on Ge(110) fabricated at 907 °C. (**a**) SEM image of the sample, showing an elongated pattern of the graphene grains. (**b**) Raman spectra for both substrate orientations with a graphene coverage of ~ 50% are shown. The spectra are derived from Raman measurements at three to six randomly selected positions on each sample after removal of the Ge background. (**c**) Position of the 2D-peak vs. position of the G-peak depicted in black and red for Ge(100) and Ge(110) for a graphene surface coverage of ca. 50%. For comparison data for a growth time of 90 min on Ge(100) are given in blue corresponding to a graphene coverage of > 100%.

In order to study the effect of substrate orientation on the graphene formation and the resulting strain properties, the TCVD experiments have been extended to Ge(110) substrates. For that purpose, graphene is fabricated until ca. 50% of the Ge surface was covered by graphene, which results in a growth time of 30 min for Ge(100) (see Figure S2(a) in SI) and 90 min for Ge(110). In Fig. 3a, the SEM image of the sample on Ge(110) is shown, where elongated dark regions related to graphene flakes are apparent on a brighter background stemming from the Ge substrate. This morphology was commonly observed in literature and attributed to the alignment of the graphene nuclei at the step edges of Ge^24^. Resulting from the anisotropy in the growth rate of armchair and zigzag edges of graphene, elongated flakes are formed^14^.

The averaged Raman spectra of the graphene on Ge(110) (in red) and Ge(100) (in black) are presented in Fig. 3b for a graphene surface coverage of ca. 50%, fabricated at the same flux and pressure values. The D-, G- and 2D-peak positions are indicated by the dashed, grey lines. The *I*_D_/*I*_G_ ratio is estimated as 0.9 and 1.2 for (100) and (110) crystal orientations, respectively. However, the 2D-peak intensity is much lower on the Ge(110) substrate. As the 2D-peak intensity was reported to be dependent on structural deformations in the graphene film^50^, we attribute the small *I*_2D_/*I*_G_ ratio to the larger amount of defects on Ge(110).

The extracted 2D- and G-peak positions are summarized in Fig. 3c for getting access to their doping and strain values. Compressive strain and p-type doping are apparent for both substrate orientations independent of surface coverage and growth time. On Ge(100), however, the strain appears to rise as the growth time and thus the surface coverage increases. Since no faceting is observed on the areas that are covered by graphene on Ge(110), we conclude that the strain must be independent of the faceting.

### Graphene synthesis on Ge(100) and Ge(110) at reduced temperatures via PECVD

**Figure 4:**
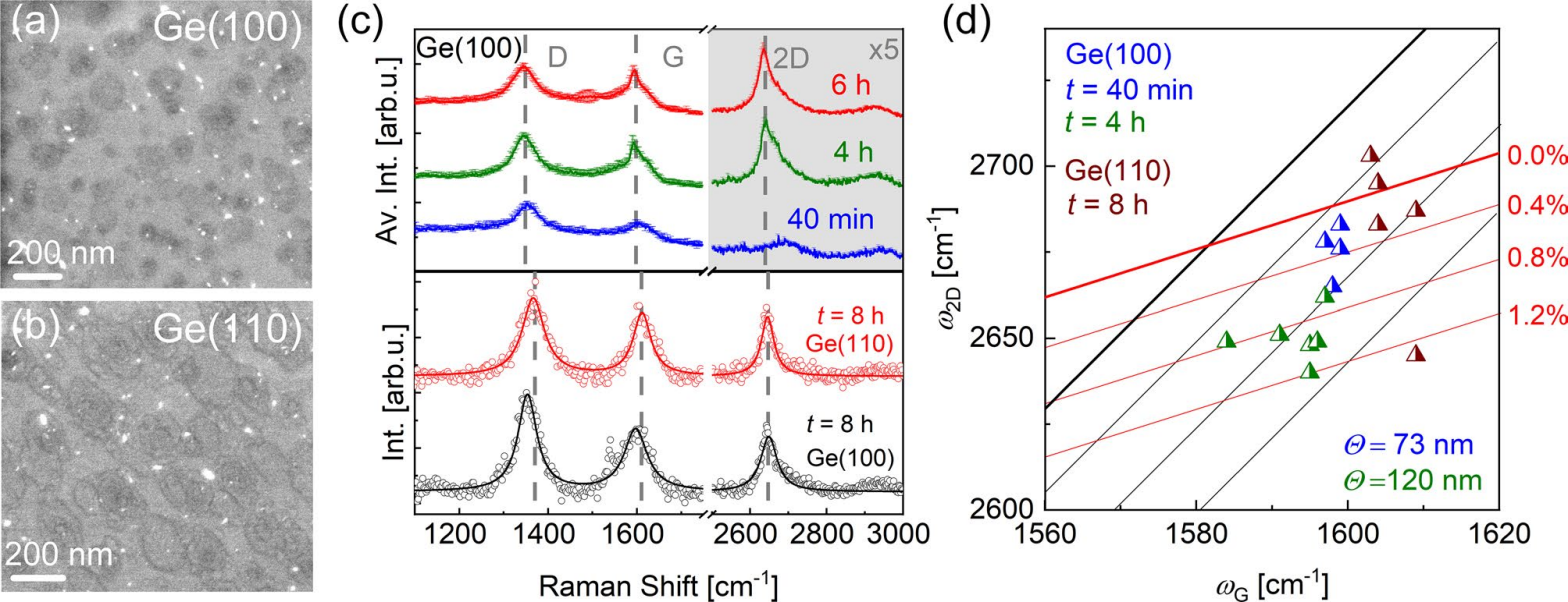
PECVD graphene on Ge(100) and Ge(110) grown at a temperature of 757 °C. In (**a**) and (**b**) the SEM images of the fabricated flakes are shown for Ge(100) and Ge(110), respectively. These samples are fabricated simultaneously for 8 h of growth time under 100 sccm CH_4_ and 200 sccm Ar flow at a plasma power of 40 W. In (**c**) Raman spectra are shown for growth times between 40 min and 6 h for graphene on Ge(100) (top). The Raman spectra are averaged over four to six spectra of randomly selected measurement points. Note that the intensity in the grey area is multiplied by 5 for an improved illustration. Raman spectra with high 2D-peak intensities are shown on the bottom of (**c**) for graphene fabricated on Ge(100) (black) and Ge(110) (red) for a growth time of 8 h. The solid lines represent Lorentzian fits to the measured data. (**d**) Position of the 2D-peak vs. G-peak position for graphene grown on Ge(100). Blue data are obtained for a growth time of 40 min, with an average grain size of 73 nm and green data are obtained for a growth time of 4 h, with an average grain size of 120 nm. Corresponding data for graphene grown on Ge(110) are shown in brown (growth time of 8 h).

In order to reduce the synthesis temperature of graphene on Ge, a plasma is ignited during the growth step to dissociate CH_4_ already in the gas phase. Graphene growth is examined on both substrate orientations at temperatures down to 757 °C. Figure 4a,b depict SEM images of samples fabricated simultaneously on Ge(100) and Ge(110) by PECVD at a growth time of 8 h under CH_4_ and Ar flows of 100 sccm and 200 sccm at a plasma power of 40 W. From the surface coverage at a given growth time it becomes obvious that the growth rate of graphene is larger on Ge(110) compared to Ge(100). A continuous increase in the surface coverage, nucleation density and grain size with growth time was observed for both orientations (see Figure S4 in SI).

To get information about quality, strain and doping levels of graphene that is synthesized via PECVD on Ge(100), Raman spectra averaged over four to six randomly chosen measurement points on the samples with growth times of 40 min (in blue), 4 h (in green) and 6 h (in red) are depicted in Fig. 4c, top. The samples were fabricated under CH_4_ and Ar flows of 3 sccm and 200 sccm at a plasma power of 40 W. The D-, G- and 2D-peaks are indicated by the dashed lines. In the early stage of growth (40 min) the Raman spectrum indicates a defective film with a very small 2D-peak. With increasing growth time, the 2D-peak intensity becomes more and more dominant, indicating an emerging long-range order in the film. The *I*_2D_/*I*_G_ ratio is calculated as 0.6 with an FWHM of 50 cm^−1^ for the 2D-peak, and a ratio *I*_D_/*I*_G_ ~ 1.3 is obtained for the sample with a growth time of 6 h.

In Fig. 4c, bottom, Raman spectra are presented in black (red) for Ge(100) (Ge(110)), which are extracted from the samples discussed in Fig. 4a,b. The *I*_2D_/*I*_G_ ratio is derived as 0.6 (0.8), and the FWHM of the 2D-peak as 39 cm^−1^ (35 cm^−1^). Although the *I*_2D_/*I*_G_ ratio is slightly lower than that of graphene fabricated by TCVD, a much sharper 2D peak is observed for graphene grown via PECVD. Therefore, it is concluded that monolayer graphene is fabricated successfully on both substrates in presence of a plasma. The *I*_D_/*I*_G_ ratio is determined to be 1.2 (1.2), which is almost two times higher than for the reference samples synthesized by TCVD. The increased number of defects is attributed to the reduced migration of carbon species on the Ge surface at low temperatures. Since on these samples also regions with a lower 2D-peak intensity are observed, we conclude that there are regions with long- (large 2D-peak intensity) and short-range order (small 2D-peak intensity). Hence, the broadening of the 2D-peak in the average Raman spectra is attributed to the overlapping signal of regions with different long-range order.

By extracting the energetic positions of the 2D- and G-peak from the Raman data, strain and doping levels of the PECVD grown graphene can be accessed. Figure 4d compares corresponding data on Ge(100) for two different growth times (40 min in blue, 4 h in green) and on Ge(110) for a growth time of 8 h (in brown). At the early stage of growth, when the average size of the flakes is 73 ± 23 nm (extracted from SEM data considering 205 flakes), the strain is about + 0.4%. With increasing growth time, the grains enlarge to 120 ± 30 nm (averaged over 186 flakes), and the tensile strain in the film is raised. Interestingly, a clear relation between the wavenumber and the intensity of the 2D-peak is found for various samples produced on Ge(100) (see Figure S6). A shift to lower wavenumbers is directly related to an increase of the 2D-peak intensity, hence to an enhanced long-range order. Apparently, the lateral order modifies the strain state of the graphene, i.e., with increasing long-range order, the tensile strain in the film increases continuously.

The observation of tensile strained graphene on Ge in case of PECVD growth is surprising, having in mind the compressive strain found in our work (see Fig. 1c) as well as in literature studies^15,23,24,51^ for graphene grown on Ge(100) via TCVD. Dabrowski et al.^22^ reported on a tensile strain of + 0.4% for TCVD graphene grown on Ge(100), which is attributed to the coalescence of small graphene domains, while its detailed origin is not described further. In our work we observed compressive and tensile strain for TCVD and PECVD grown samples, respectively, regardless the growth parameters and substrate orientation. Our findings may act as a first hint of a different growth mechanism of graphene by PECVD as compared to TCVD.

### Mechanism of graphene formation and strain development in TCVD and PECVD on Ge

Since the graphene flake size is around 100 nm, it is not possible to extract spatially resolved information via Raman spectroscopy with a laser spot size of 0.5 µm. Therefore, the data base that is required for the development of a suitable growth model is expanded and the surface topography and the electronic properties are examined by Kelvin probe force microscopy. During the measurements, topography and contact potential difference (CPD) are collected simultaneously. The CPD is proportional to the work function difference between the tip and the sample. For a given tip, the CPD along the sample surface can be extracted with a spatial resolution of < 20 nm and a sensitivity of < 20 mV^52^. This allows us to differentiate the work function of the graphene flakes and their surroundings. Note that both the tip and the measurement conditions are kept constant during the following measurements and are therefore directly comparable to each other.

**Figure 5:**
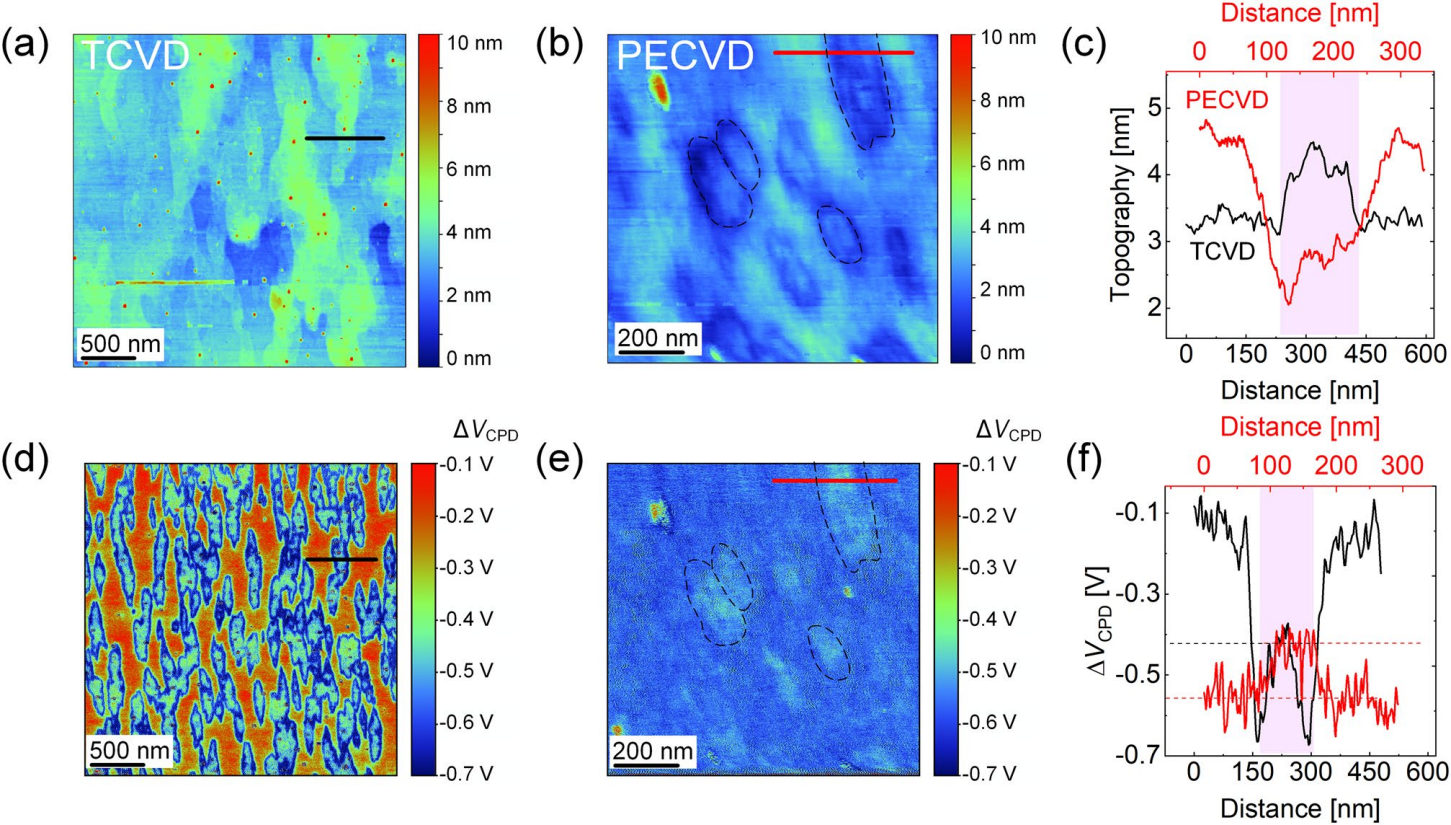
Topography and Δ*V*_CPD_ measurements for TCVD and PECVD grown graphene on Ge(110). In (**a**) the topography map of the reference TCVD sample grown without plasma is shown. Graphene flakes are indicated by the green areas. Akin to that in (**b**) the topography image of the sample is shown, which is fabricated in presence of a plasma. The black dotted lines emphasize graphene flakes. (**c**) Topography profiles extracted at the positions marked by black and red lines in (**a**) and (**b**), respectively. Black and red data are related to samples grown with and without plasma, respectively. The flake area is indicated by the pink region. (**d**) Δ*V*_CPD_ map for the TCVD sample indicating elongated graphene flake formation. Similarly, in (**e**) the Δ*V*_CPD_ map is shown for the PECVD grown graphene with the flakes indicated by black dashed lines. In (**f**) Δ*V*_CPD_ profiles are depicted, which are extracted from the maps and indicated correspondingly as black and red lines in (**d**) and (**e**).

The atomic force microscopy (AFM) topography map of a sample fabricated without plasma on Ge(110) is shown in Fig. 5a. The SEM image of this TCVD grown sample has already been presented in Fig. 3a, on which graphene flake formation was apparent. Similarly, elongated structures are observed in the topography map, indicated by the green colour. For comparison, the topography of graphene grown by PECVD on Ge(110) at 757 °C with a CH_4_/H_2_ flux ratio of 3 sccm/100 sccm and a growth time of 2 h at a plasma power of 100 W is presented in Fig. 5b using the same height scale. Graphene flakes are indicated by the dashed, black lines. Note, that opposite to Fig. 5a, the graphene flakes (dark blue) are apparently surrounded by elevated regions (light blue). Furthermore, in the center of some flakes a slightly elevated topography is apparent, which we attribute to multilayer islands. This is supported by SEM images (see Figure S5 in the SI), where dark grains with round to hexagonal morphology and sizes in the order of 100 nm are visible on the surface, and multilayer islands can be seen in the center of some graphene flakes (indicated by white arrows in Figure S5).

In order to quantify the height profile, line scans across selected graphene flakes are extracted, indicated by the black and red lines in Fig. 5a,b, respectively. The topography profiles are given in Fig. 5c for TCVD (PECVD) grown graphene in black (red). The graphene flake region is indicated by the pink shaded area. The graphene flakes that are fabricated without plasma are ~ 0.8 nm above the substrate area. This value confirms that the flakes are of monolayer nature^53^. The height of the PECVD graphene regions is determined as 0.9 ± 0.3 nm by averaging over four grains with reference to the lowest measured point. Around the flakes on the other hand, the height increases by about 2 ± 0.2 nm.

Simultaneously to the topography measurements, the CPD voltage is collected over the sample for getting access to the local work function. As a reference, the raw Ge(110) substrate is measured by the same tip prior to each measurement, and the contact potential difference between the graphene covered samples and the raw Ge substrate is extracted (Δ*V*_CPD_ = *V*_CPD,graphene_ − *V*_CPD,GeRAW_). In Fig. 5d, a Δ*V*_CPD_ map is shown for the graphene grown by TCVD. The graphene areas are represented by the blue/green colours, indicating the elongated shape of the graphene flakes. The Ge substrate surface, on the other hand, is characterized by the red coloured areas, i.e., by significantly reduced values of Δ*V*_CPD_ with respect to the raw Ge(110) wafer.

The Δ*V*_CPD_ map of the sample that is fabricated in presence of a plasma is shown in Fig. 5e. The flakes are represented by the blue/green colour, indicating a similar contact potential difference as graphene flakes grown via TCVD (see Fig. 5d). However, between the flakes a homogenous area with an enhanced Δ*V*_CPD_ is apparent, indicated by the dark blue colour. No regions with red colour, i.e., low Δ*V*_CPD_ values, are observed on this sample. Hence, it is concluded that the Ge wafer is fully covered after the plasma process.

In the next step, Δ*V*_CPD_ profiles are extracted at the flake region (pink shaded area, see Fig. 5f) similar to the topography measurements (compare Fig. 5c). The samples grown without and with plasma are represented in black and red colour, respectively. Apparently, the graphene flake region is characterized by Δ*V*_CPD_ ≈ − 0.43 V, regardless the fabrication method. Surprisingly, Δ*V*_CPD_ is strongly different outside of the graphene flakes for TCVD and PECVD prepared samples. I.e., Δ*V*_CPD_ ≈ − 0.56 V for the surroundings in the PECVD grown layer, whereas Δ*V*_CPD_ approaches ≈ − 0.15 V in case of the TCVD grown sample.

In order to quantify the corresponding work functions, a reference gold film is measured with the same tip and a *V*_CPD_ = 0.53 V is derived. By assuming a work function of 4.8 eV^54^ for the gold reference, the work function of the tip is determined as *Φ*_Tip_ = 5.33 eV. With the measured CPD of *V*_CPD,GeRAW_ = 0.68 V between tip and the as-received raw Ge wafer (assumably with a native oxide layer on top), the work function of the raw Ge(110) is estimated as *Φ*_Ge,raw_ = *Φ*_Tip_ − e∙*V*_CPD_ = 4.65 eV, which agrees well with the literature value of 4.67 eV^55^. The work function of the graphene grains is determined as *Φ*_graphene_ = *Φ*_Ge,raw_ − e∙Δ*V*_CPD_ ≈ 5.1 eV, regardless the fabrication method. The work function of pristine graphene on Si/SiO_2_ is reported between 4.2 eV^56^ up to 5.11 eV^57^ in literature, and is shown to increase in presence of p-type doping^58^. The relatively large work function of our graphene thus indicates p-type doping, agreeing with our Raman data.

A Δ*V*_CPD_ of ~  − 0.1 V … − 0.2 V is derived for the substrate area around the graphene flakes for TCVD (see Fig. 5e,f), which indicates a work function of 4.75 eV… 4.85 eV for the Ge wafer after the CVD process. We believe the slight variation of the work function of the substrate area after TCVD growth as compared to the raw Ge wafer is due to the melting during high temperature processing and/or oxidation of the Ge afterwards. The Δ*V*_CPD_ ≈ − 0.56 V extracted for the surrounding of the graphene flakes in PECVD (see Fig. 5e,f), however, corresponds to a work function of ~ 5.2 eV. This value is close to theoretical values of amorphous carbon^59^, thus indicating the formation of a defective carbon layer around the crystalline graphene flakes in PECVD, similar to what is reported in literature for PECVD grown graphene on Cu^60^.

Regarding these data, it is obvious that in case of TCVD, isolated monolayer graphene islands of elongated shape are formed on top of the Ge(110) surface. In contrast, the graphene flakes grown by PECVD are surrounded by a 2 ± 0.2 nm thick defective carbon film covering the Ge surface. The same trend is also observed for samples that are fabricated on Ge(100) (see Figure S7 in SI) under the same conditions as on Ge(110). Regarding the similar work function and height values regardless the fabrication method, the PECVD graphene is concluded to be of monolayer nature.

**Figure 6:**
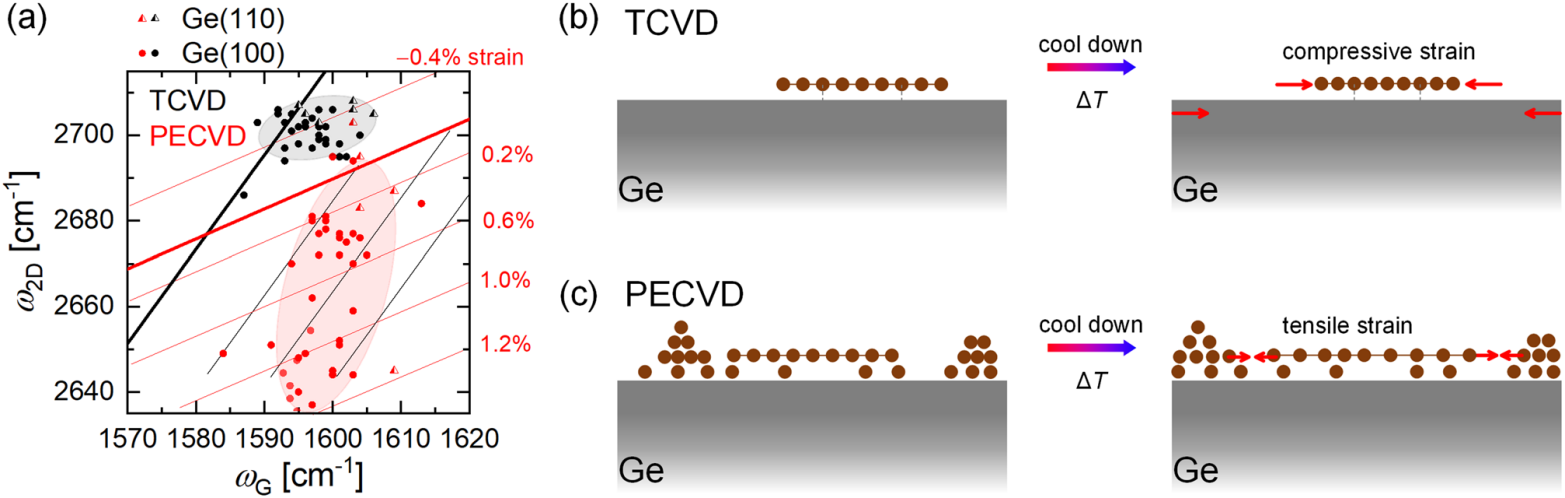
Strain properties and model idea for the growth and the related origin of strain formation in graphene films. In (**a**) the wavenumber of the 2D-peak is plotted vs. the G-peak position for TCVD in black and PECVD in red colour. The square and triangle symbols represent the Ge(100) and Ge(110) wafer. The shaded areas illustrate the accumulation of the data and serve as guide to the eye. A sketch of our model idea explaining the origin of the different strain states in graphene films, which are fabricated by TCVD and PECVD, are shown in (**b**) and (**c**), respectively.

Combining these results with the Raman data discussed above, a mechanism is suggested to explain the graphene formation and the related origin of the observed strain. Figure 6a summarizes the relation between the wavenumbers of the 2D- and the G-peak, i.e., *ω*_2D_ vs. *ω*_G_, for a wide variety of samples that are fabricated via TCVD (black) and PECVD (red) on Ge(100) and Ge(110) as indicated by squares and triangles, respectively. Independent of the substrate orientation or the growth parameters, a quite homogenous, compressive strain is observed for graphene fabricated by TCVD within our set of experiments. The samples in addition show p-type doping agreeing with most literature^15,51^. Graphene films, which are synthesized at reduced temperatures in presence of a plasma, on the other hand, exhibit a pronounced tensile strain up to + 1.2%. Considering the scattering of the data (size of the shaded areas), the PECVD samples apparently vary regarding strain and doping properties, indicating deviations in the long-range order and/or defect formation across the sample.

Our analysis finally allows us to propose a model idea for the graphene growth on Ge, explaining the different strain states found in TCVD and PECVD. In Fig. 6b,c, the C atoms are presented as brown dots and graphene flakes are illustrated by a periodic arrangement of C atoms. The red arrows indicate the direction of lattice expansion / compression upon the cooling process after CVD.

In TCVD, the Ge wafer is expected to contract during cool down, while the graphene layer tends to expand as indicated by the different signs of the thermal expansion coefficients. As in TCVD the strain of the graphene is compressive, we assume a strong interaction between the graphene and Ge wafer. This interaction forces graphene to shrink during cooling. After delamination, this graphene–Ge interaction disappears, and the graphene film relaxes. This leads to a reduced strain in graphene transferred on a Si/SiO_2_ substrate. In PECVD, in contrast, a defective carbon film is located around the crystalline flakes and probably also beneath them. As a result, the interaction between the graphene and Ge may be weakened. During the cooling process, the graphene areas expand, and the edge carbon atoms from the crystalline flakes and the surrounding defective carbon layer become closer, potentially forming bonds in order to saturate the existing dangling bonds. The attractive forces between the graphene and the defective regions would thus induce the tensile strain in the graphene areas, in agreement with our findings.

## Conclusion

We demonstrate graphene fabrication on Ge wafers with (100) and (110) crystal orientation by plasma-enhanced CVD at reduced temperatures. In reference experiments, where the plasma is switched off, monolayer graphene with a *I*_2D_/*I*_G_ ratio of 3.9 is synthesized at temperatures close to the melting point of Ge, and a sheet resistance of 2.5 kΩ/sq. is determined. The resulting film exhibits a compressive strain of − 0.4% regardless the substrate orientation and faceting, which is related to the strong interaction between graphene and Ge substrate during the cooling after growth. In presence of a plasma, the growth temperature is reduced to 757 °C—the lowest growth temperature for graphene on Ge hitherto—and round to hexagonal and elongated flakes are observed on Ge(100) and Ge(110), respectively. With increasing growth time the grain size of graphene increases and a pronounced 2D-peak emerges indicating the long-range order in the film. A tensile strain is monitored for the PECVD grown films. Combining our findings from Raman spectroscopy with Kelvin probe force measurements we demonstrate that in case of TCVD, isolated graphene grains are formed on the substrate, merging to a film with growth time, whereas in case of PECVD, graphene flakes are surrounded by a defective carbon film.

## Methods

The Ge(100) wafer is grown epitaxially on a Si wafer with a film thickness of 2 µm in order to avoid any Si diffusion to the surface during graphene growth. The Ge(110) wafer, on the other hand, is a pure Ge wafer. Prior to the process, the wafers are cut into pieces with one cm^2^ in size and cleaned in boiling acetone and ethyl alcohol for 2 min. The substrates are then flushed by isopropyl alcohol and blown dry under a stream of nitrogen.

CVD growth of graphene on Ge is established in a 4″ *Black Magic* system from *Aixtron Ltd*. Prior to the graphene synthesis, the wafer was loaded to the chamber and the temperature was increased until the sample surface begins to melt at a chamber pressure of 750 mbar. The processing temperature was adjusted roughly 10 K for Ge(100) or 30 K for Ge(110) below the noted thermocouple temperature, at which the substrate started to melt. Note that since the melting temperature is defined by visual analysis, there is an inevitable error in the determination of the substrate temperature. The given temperature values in the manuscript were calculated from the melting temperature of 937 °C.

In the plasma enhanced method, a pulsed-DC plasma with a pulse frequency of 10 kHz and 1 µs of reverse time is ignited in the same chamber during the growth process. The plasma power is varied between 40 and 100 W and the external voltage is regulated dependent on the current flowing to the electrodes. To shield the electrical field between the plasma sheath and the substrate, a Ni foil was folded into an inverted cup, which served as a Faraday cage. Note that for each process a fresh foil was used. Details of the process and the sacrificial foil are discussed in more detail elsewhere^60^. The chamber pressure was adjusted to 4 mbar at a constant temperature of 757 °C. As precursor gas CH_4_ is used at varied flow rate between 3 and 100 sccm. The plasma atmosphere is realized by either H_2_ or Ar flow. The process parameters of the individual samples are indicated in the manuscript.

The Raman spectra were taken with a 532 nm incident laser wavelength in a *NTEGRA Spectra System* from *NT-MDT*. In order to transfer the graphene film, Poly(methyl methacrylate) (PMMA) is spin-coated to protect the film during the standard electrochemical delamination method^61^ in a NaOH bath against a platinum electrode at an external voltage between 3 and 4 V. The graphene film is then separated by the evolving H_2_ gas between graphene and Ge and fished out of the H_2_O bath by a Si/SiO_2_ substrate (285 nm of SiO_2_). The PMMA was removed by leaving the sample ca 1.5 h in the acetone bath. For TLM measurements, electrodes with 15 nm Ti and 85 nm Au were evaporated onto the graphene film by a shadow mask method. The spacing between the contact pads (*d*) are 20 µm, 40 µm, 60 µm, 80 µm, 100 µm and 120 µm for the first and 20 µm, 80 µm, 140 µm, 200 µm, 260 µm and 320 µm for the second structure with a width (*W*) of 400 µm for one contact pad. Electrical properties of the film were determined from the slope of the total resistance at a source-drain voltage of 0.1 V vs. *d*/*W*.

AFM and Kelvin probe force microscopy measurements were performed by a *Veeco*/*Bruker Innova Atomic Force Microscope* directly on the Ge wafer. The Ge wafer was grounded and the contact potential difference (*V*_CPD_) between the substrate and the *ElektriMulti75-G* tip with a Pt (95%)/Ir (5%) coating is measured and the work function is calculated with the relation *V*_CPD_ = (*Φ*_Tip_ − *Φ*_Sample_)/e. Prior to and after each measurement the raw Ge wafer of the corresponding orientation was measured as a reference and the ∆*V*_CPD_ is extracted relative to the reference.

The morphology of the grown samples was characterized by scanning electron microscopy (ZEISS Supra 25) with an in-lens and an Everhart–Thornley detector. The operation parameters were a primary electron energy of 1 keV and working distance of 4–5 mm.

## Supplementary information

Supplementary Information.
