# Supplementary material for: Direct growth of graphene on Ge(100) and Ge(110) via thermal and plasma enhanced CVD
Source: Sci Rep. 2020 Jul 31;10:12938. doi: 10.1038/s41598-020-69846-7 (PMC7395096; doi:10.1038/s41598-020-69846-7)
Supplement: Supplementary file 1 — Supplementary Information. [file 41598_2020_69846_MOESM1_ESM.pdf]

Direct growth of graphene on Ge(100) and Ge(110) via thermal and plasma enhanced CVD

Bilge Bekdüz, Umut Kaya, Moritz Langer, Wolfgang Mertin and Gerd Bacher

Werkstoffe der Elektrotechnik and CENIDE, Universität Duisburg-Essen, 47057 Duisburg, Germany

-Supporting Information-

Table of content

Figure S1: Topography profiles extracted from AFM measurements.

Figure S2: Graphene growth as a function of growth time on Ge(100) by TCVD.

Figure S3: Raman spectra of graphene fabricated on Ge(100) by TCVD by varying the amount of CH_4 and H_2.

Figure S4: Graphene coverage in PECVD as a function of growth time.

Figure S5: Multilayer islands on graphene fabricated by PECVD on Ge(100).

Figure S6: Position of the 2D-peak as a function of the 2D-peak intensity.

Figure S7: Topography and ΔV_{CPD} maps for PECVD grown graphene on Ge(100).

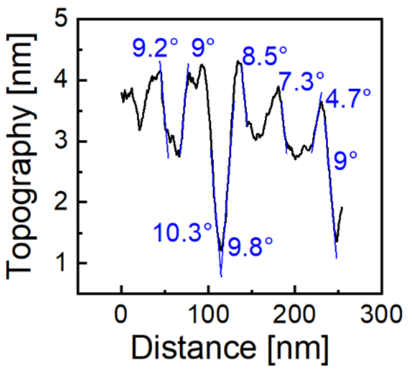

Figure S1. Topography profiles extracted from AFM measurements at randomly selected positions of the sample presented in Figure 1(a) in the manuscript. An average angle of 8.5° is extracted, which corresponds to {107} facets according to McElhinny et al., Surface Science 647 (2016) 90–95.

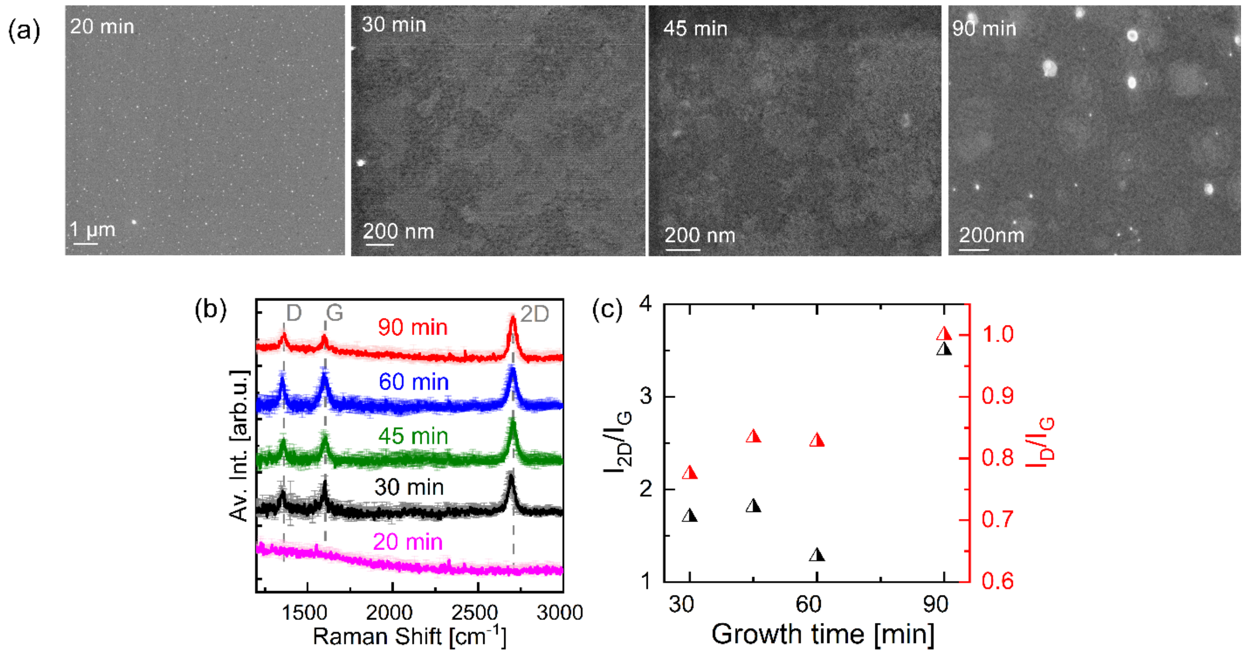

Figure S2 (a) SEM images of samples fabricated at different growth times between 20 min to 90 min. (b) Average Raman spectra shown for different durations of the growth process. These data are extracted from three randomly selected positions on the samples presented in the manuscript in Figure 1(c) after removing the background signal. (c) I_{2D}/I_G (in black) and I_D/I_G (in red) ratio plotted vs. growth time. These values are extracted from the average Raman spectra given in (b).

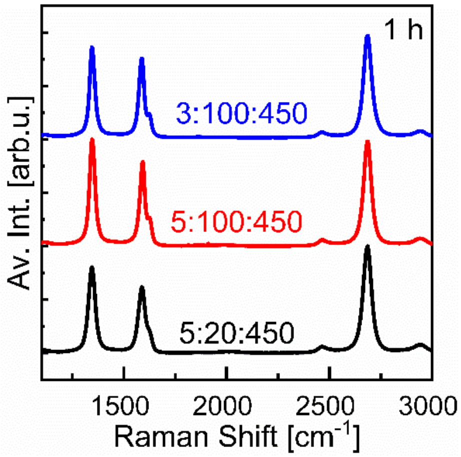

Figure S3. Raman spectra for samples fabricated under CH_4:H_2:Ar flows of 5:20:450, 5:100:450 and 3:100:450, which are averaged over 224, 224 and 402 measurement points from an area of 10 µm x 10 µm, 7 µm x 7 µm and 10 µm x 10 µm after transferring the films onto SiO_2/Si substrates. The growth time was kept constant at 1 hour. Notice that the spectrum in black represents the sample shown in Figure 2(c), on which the sheet resistance is measured.

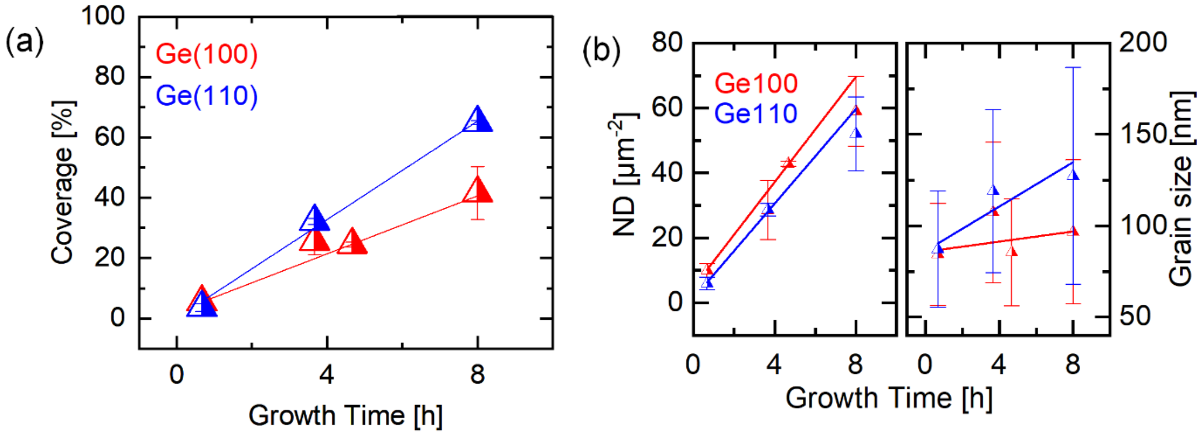

Figure S4. (a) Graphene coverage and (b) nucleation density and grain size plotted as a function of growth time in red for Ge(100) and in blue for Ge(110). The samples are fabricated by PECVD with a methane and argon flow of 100 sccm and 200 sccm, respectively, and a plasma power of 40 W.

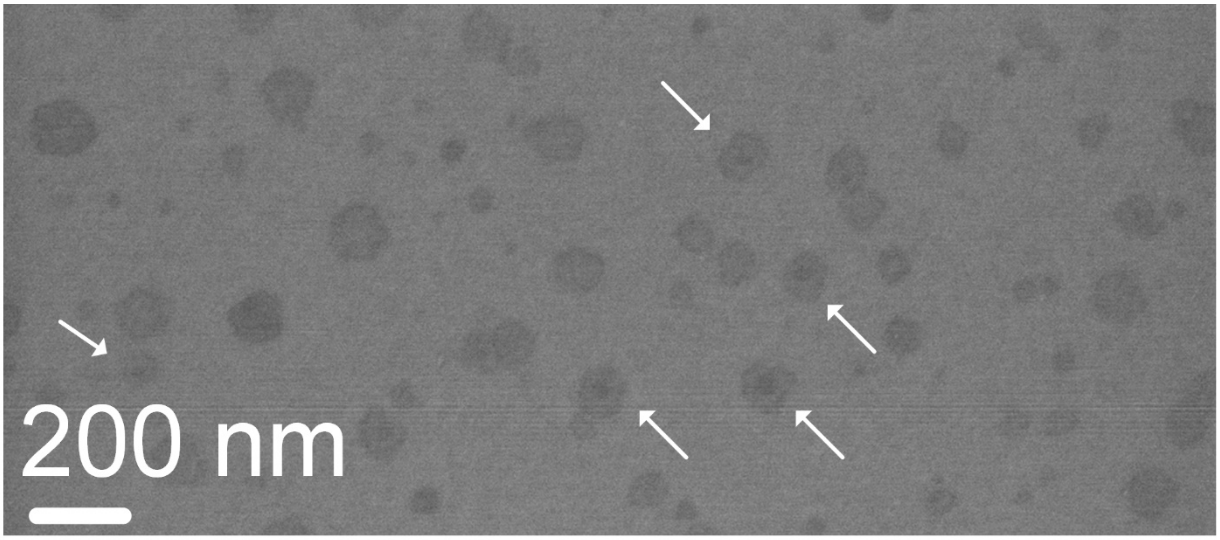

Figure S5. Multilayer formation in the center of graphene flakes. Graphene is fabricated on Ge(100) at a temperature of 757°C by PECVD for a growth time of 40 min under CH_4 and Ar flows of 100 sccm and 200 sccm at a plasma power of 40 W. Dark grains with round to hexagonal morphology and sizes in the order of 100 nm are visible on the surface. Multilayer islands in the center of some graphene flakes are indicated by white arrows.

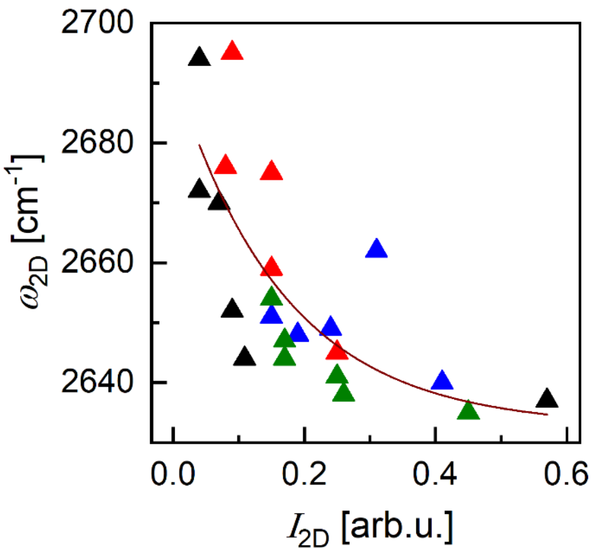

Figure S6. 2D-peak position as a function of 2D-peak intensity. The relation between the 2D-peak position and the intensity is shown for four different samples, indicated by different colors. With emerging 2D-peak and thus increasing long-range order, the 2D-peak shifts down in energy, which translates into an increase in the tensile strain. The brown solid line serves as a guide to the eye.

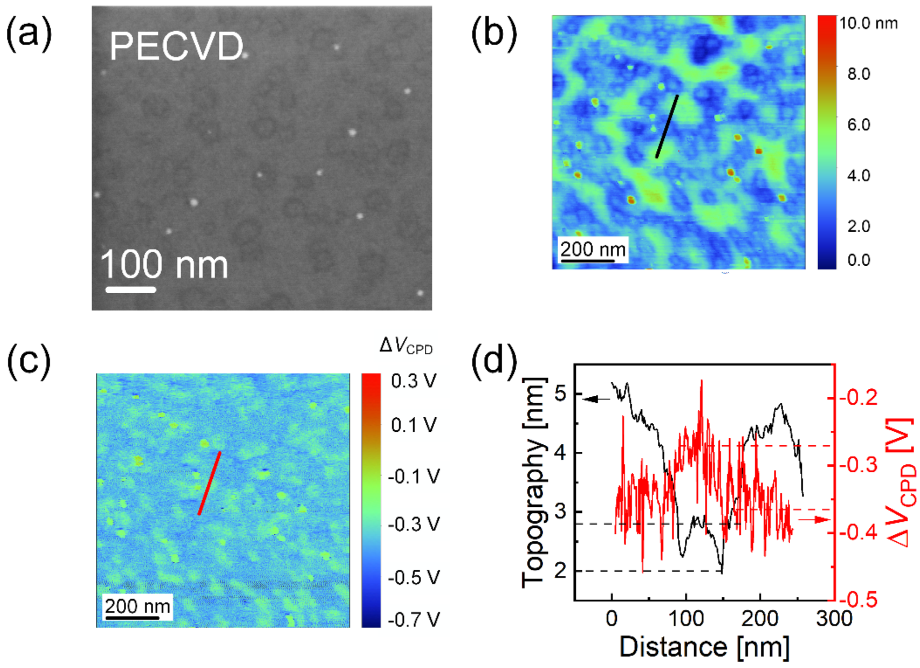

Figure S7. Topography and ΔV_{CPD} measurements of PECVD grown graphene on Ge(100). (a) SEM image for a sample that is fabricated simultaneously with the one presented in Fig 5(b). Similar to what is discussed in Fig 5, an elevated topography is apparent around the flakes. In (c), the ΔV_{CPD} map is shown. In (d), a topography and a ΔV_{CPD} profile are shown exemplarily for one flake (indicated by the black and red lines shown in the figure). The height of the surrounding is about 2 nm above the flake region.
